# Current and emerging artificial intelligence applications in chest imaging: a pediatric perspective

**DOI:** 10.1007/s00247-021-05146-0

**Published:** 2021-09-01

**Authors:** Steven Schalekamp, Willemijn M. Klein, Kicky G. van Leeuwen

**Affiliations:** Department of Medical Imaging, Radboud University Medical Center, P.O. Box 9101, 6500 HB Nijmegen, The Netherlands

**Keywords:** Artificial intelligence, Chest radiography, Children, Computed tomography, Pediatric radiology, Thorax

## Abstract

Artificial intelligence (AI) applications for chest radiography and chest CT are among the most developed applications in radiology. More than 40 certified AI products are available for chest radiography or chest CT. These AI products cover a wide range of abnormalities, including pneumonia, pneumothorax and lung cancer. Most applications are aimed at detecting disease, complemented by products that characterize or quantify tissue. At present, none of the thoracic AI products is specifically designed for the pediatric population. However, some products developed to detect tuberculosis in adults are also applicable to children. Software is under development to detect early changes of cystic fibrosis on chest CT, which could be an interesting application for pediatric radiology. In this review, we give an overview of current AI products in thoracic radiology and cover recent literature about AI in chest radiography, with a focus on pediatric radiology. We also discuss possible pediatric applications.

## Introduction

Chest radiography is one of the most commonly requested examinations in the workup of pediatric patients under suspicion of having a variety of diseases including pneumonia, tuberculosis and pneumothorax [1]. Chest radiographs are also commonly acquired to confirm the location of lines and tubes, or to surveil disease including oncologic disease. Chest CT is less commonly used in the pediatric population but can be helpful for assessing children with bronchial disease, infectious disease or interstitial lung disease.

Artificial intelligence (AI) software for chest imaging is being widely studied within radiology. To date this has resulted in more than 40 CE (Conformité Européenne)-marked commercial software packages from more than 20 vendors being available for clinical use in CT and conventional radiography of the thorax [2]. A wide range of abnormalities is covered by AI, in both chest radiography and chest CT. Most of the current AI products have been developed for the adult population, but they might be applicable to the pediatric population as well.

In this review, we discuss commercially available AI products for thoracic radiology. Because most of the current AI applications might not be specifically applicable to the pediatric population, we also discuss recent literature showing the potential for AI use in pediatric chest imaging. We emphasize the applications of AI in chest radiography rather than chest CT because radiography applications are of more interest in the pediatric population.

## Current landscape of artificial intelligence products in thoracic radiology

**Fig. 1:**
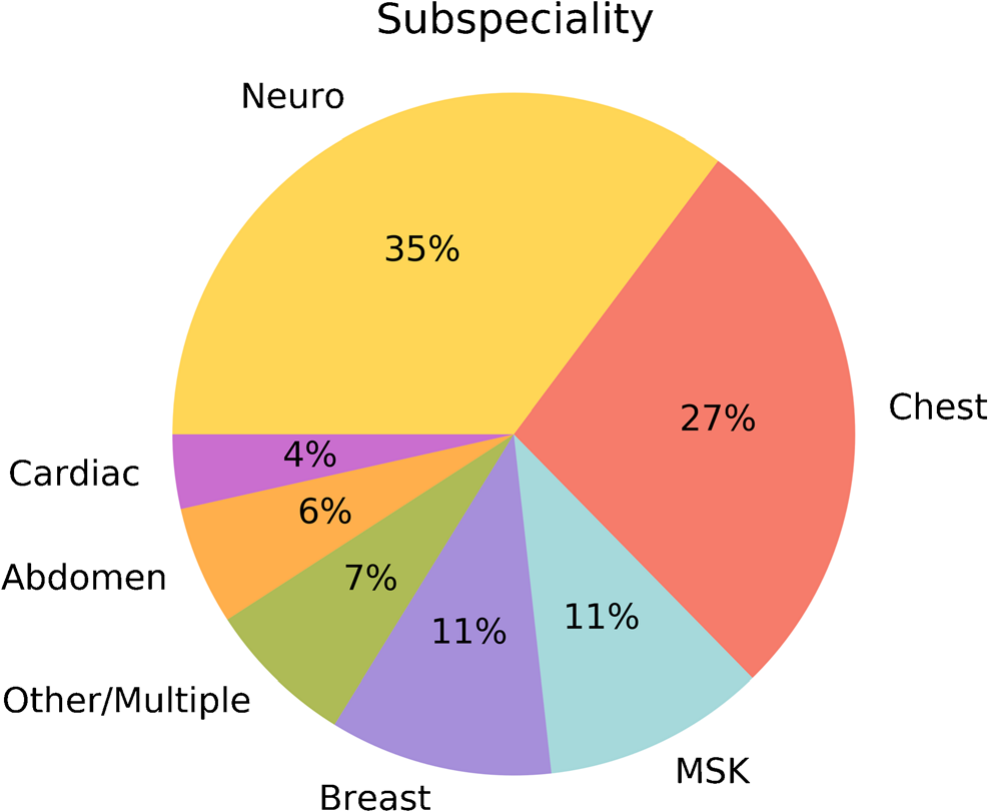
Pie chart shows distribution by radiologic subspecialty of the 144 artificial intelligence (AI) products certified for image analysis in radiology at the time of this report [2]. *MSK* musculoskeletal, *Neuro* neurologic

More than 40 AI applications are CE marked for thoracic radiology. This is the largest number of AI applications in radiology by subspecialty, after neuroradiology (Fig. 1) [2, 3]. Half of the products are developed for the analysis of chest radiography, and half for chest CT. No specific products have been developed and certified for the pediatric population (Table 1) [4–29]. However, some products might be applicable to the pediatric population, as well. Current research per chest imaging modality is discussed next. In this review we emphasize the diagnostic applications, focusing on currently available commercial AI systems. Post-processing AI applications are not considered in this review.

**Table 1:** Overview of current CE (Conformité Européenne)-certified artificial intelligence (AI) software in chest radiography and chest CT

					Types of disease targeted^b^									
Company name	Product name	Modality	Single or multiple abnormalities	Peer-reviewed journal papers on performance^a^	Lung cancer/nodules	Pulmonary embolism	Pneumothorax	Tuberculosis	Interstitial lung disease	Emphysema	Obstructive lung disease	Pneumonia	Rib fracture	Signs of cardiac failure
Arterys (San Francisco, CA)	Chest | MSK AI	CR	Multiple	No	x		x						x	
Behold.ai (London, UK)	Red Dot	CR	Multiple	No			x							
GE Healthcare (Chicago, IL)	Critical Care Suite	CR	Single	No			x							
Infervision (Beijing, China)	InferRead DR Chest	CR	Multiple	No	x		x	x				x	x	x
JLK Inc. (Seoul, Korea)	JLD-02K	CR	Single	No	x									
Lunit (Seoul, Korea)	Lunit INSIGHT CXR	CR	Multiple	Yes [4–6]	x		x	x				x		x
Oxipit (Vilnius, Lithuania)	ChestEye CAD	CR	Multiple	No	x		x	x	x	x	x	x		x
QUIBIM (Valencia, Spain)	Chest X-Ray Classifier	CR	Multiple	Yes [7]	x		x		x	x		x		x
Qure.ai (Mumbai, India)	qXR	CR	Multiple	Yes [8–10]	x		x	x				x	x	x
Riverain Technologies (Miamisburg, OH)	ClearRead Xray – Compare	CR	Single	No	x									
Riverain Technologies	ClearRead Xray – Bone Suppress	CR	Single	Yes [11–13]	x									
Riverain Technologies	ClearRead Xray – Detect	CR	Single	Yes [14, 15]	x									
Riverain Technologies	ClearRead XRay – Confirm^c^	CR	Single	No										
Samsung Electronics (Seoul, Korea)	Auto Lung Nodule Detection	CR	Single	Yes [16]	x									
Siemens Healthineers (Erlangen, Germany)	AI-Rad Companion Chest X-ray	CR	Multiple	No	x		x					x		x
Thirona (Nijmegen, the Netherlands)	CAD4TB	CR	Single	Yes [17]				x						
Thirona	CAD4COVID-XRay	CR	Single	Yes [18]								x		
VUNO (Seoul, Korea)	VUNO Med-Chest X-ray	CR	Multiple	Yes [19]	x		x		x			x		x
Zebra Medical Vision (Kibbutz Shefayim, Israel)	Triage Pneumothorax	CR	Single	No			x							
Zebra Medical Vision	Triage Pleural Effusion	CR	Single	No										x
Aidence (Amsterdam, the Netherlands)	Veye Chest	CT	Single	No	x									
Aidoc (Tel Aviv, Israel)	Pulmonary embolism	CT	Single	No		x								
Arterys	Lung AI	CT	Single	No	x									
Icometrix (Leuven, Belgium)	icolung	CT	Single	No								x		
Imbio (Minneapolis, MN)	Lung Texture Analysis	CT	Multiple	Yes [20, 21]					x					
Imbio	Lung Density Analysis – Functional	CT	Multiple	Yes [22, 23]							x			
Infervision	InferRead CT Pneumonia	CT	Single	No								x		
Infervision	InferRead CT Lung	CT	Single	No	x									
JLK Inc.	JLD-0xK	CT	Single	No	x									
MeVis Medical Solutions AG (Bremen, Germany)	Veolity	CT	Single	Yes [24–26]	x									
Mindshare Medical (Seattle, WA)	RevealAI-Lung	CT	Single	No	x									
QUIBIM	Lung densities	CT	Multiple	No						x	x			
Riverain Technologies	ClearRead CT – Detect	CT	Single	Yes [27, 28]	x									
Riverain Technologies	ClearRead CT – Vessel Suppress	CT	Single	Yes [27, 28]	x									
Riverain Technologies	ClearRead CT – Compare	CT	Single	No	x									
Siemens Healthineers	AI-Rad Companion CT	CT	Multiple	Yes [29]	x							x		
Thirona	CAD4COVID-CT	CT	Single	No								x		
Thirona	LungQ	CT	Multiple	No						x	x			
VIDA (Coralville, IA)	VIDA Insights	CT	Multiple	No					x	x	x	x		
VUNO	VUNO Med-LungCT AI	CT	Single	No	x									

*CAD* computer-aided detection, *CR* chest radiography, *CT* computed tomography, *CXR* chest radiography, *MSK* musculoskeletal

^a^Per product, current available peer-reviewed literature on performance is given

^b^Some products might also detect other abnormalities than listed in this table. Up-to-date information on the products can be found on www.AIforRadiology.com

^c^Image enhancement product that enables better visualization of lines and tubes

### Chest radiography

**Fig. 2:**
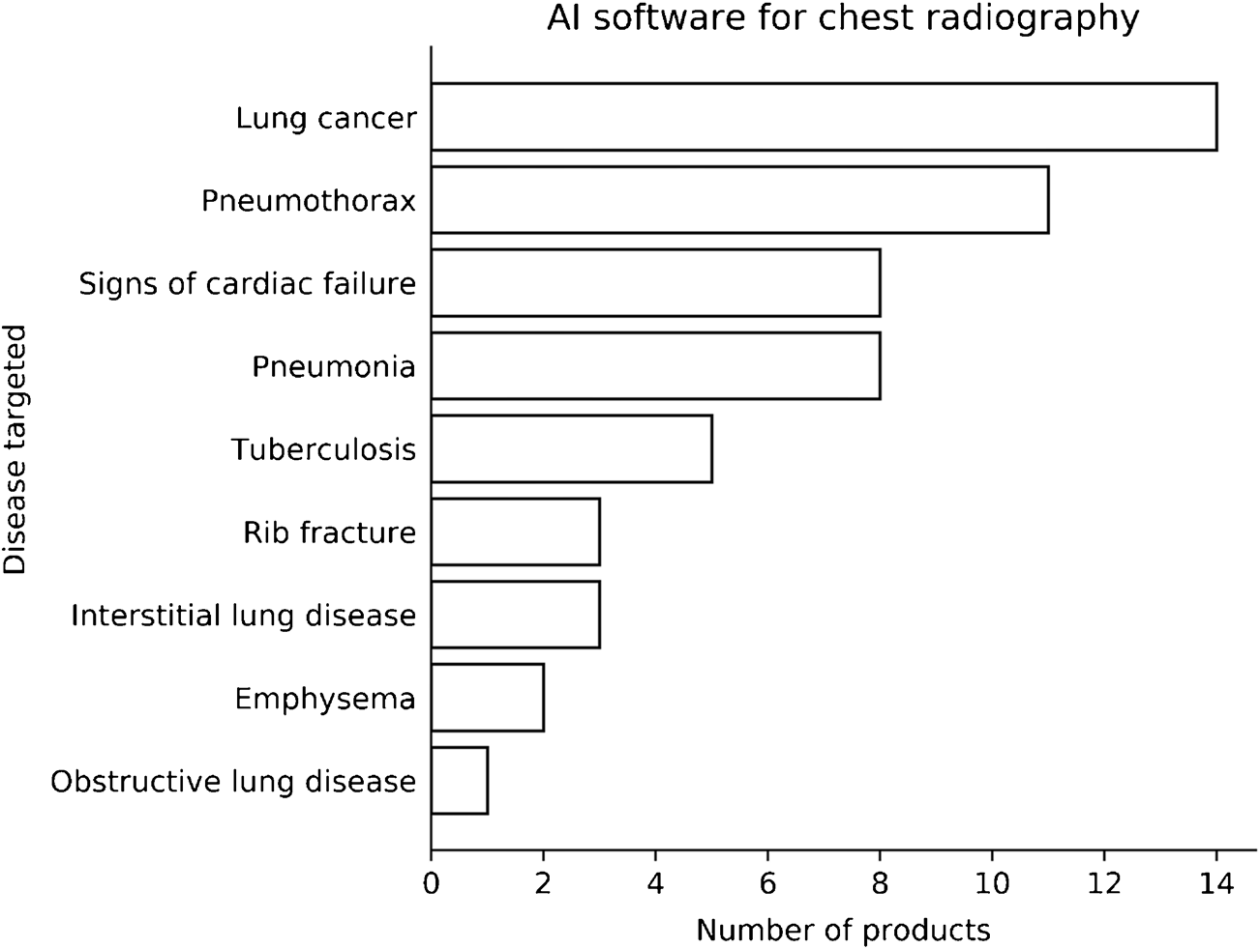
Bar chart shows artificial intelligence (AI) products available for chest radiographs at the time of this report [2]

The first AI software products, or computer-aided detection software, for conventional chest radiography were designed to detect lung nodules with the goal to reduce missed lung cancers. These tools were then extended to detect other abnormalities on chest radiographs, such as pneumonia, pneumothorax and rib fractures (Fig. 2). A subset of applications is intended for triage, prereading images and flagging images on which urgent findings were detected by the AI system.

#### Lung nodules

Automated lung nodule detection is the most studied application for chest radiography. Research that investigated the performance of lung nodule detection in chest radiographs started decades ago. With few publicly available datasets, systems were hard to compare. Steady improvement has been shown on the Japanese Society of Radiological Technology (JSRT) dataset consisting of chest radiographs with and without lung cancer, which can be used for validation purposes. First studies from 1999 reached a sensitivity of 35% at 6 false positives per image [30]; by 2014, this improved to a sensitivity of 75% at 0.5 false positives per image on this dataset [31]. Recently developed AI systems that incorporated deep learning have not tested their performances on the JSRT data but do report improved performances up to a sensitivity of about 80% at only 0.05 false positives per image [4].

For lung nodule detection, performance of these AI systems is similar or even surpasses the performance of radiologists. In a study from Nam et al. [4], a deep learning system that was trained on more than 40,000 chest radiographs showed in receiver operating characteristics (ROC) analysis an area under the curve (AUC) of 0.92–0.99 (depending on the validation set) for the detection of lung nodules with an average size ranging 20–40 mm. AI performance was similar or significantly better than that of the participating thoracic radiologists in this study. Most readers had improved performance when aided by the deep learning system.

Another recent study, by Yoo et al. [32], assessed the performance of their algorithm on chest radiographic screening data from the National Lung Screening Trial. On the baseline screening data, consisting of 5,485 radiographs, the algorithm reached a sensitivity of 94.1% and a specificity of 83.3% for detecting malignant pulmonary nodules.

Up to now, 11 certified products have been made available to help detect pulmonary nodules in chest radiographs. The developed systems have been trained on adult data, often older people with a high risk of lung cancer. However, the spectrum of abnormalities in the pediatric chest is different from that in adults. In the pediatric population, it would be more helpful to detect pulmonary metastases instead of potential lung cancers, which is relevant, for example, in children with osteosarcoma. However, such systems that aim to detect pulmonary metastases specifically are not yet available, and current systems trained on lung cancer data probably have a lower performance for the detection of pulmonary metastases. Furthermore, the pediatric chest is quite different from the adult chest with respect to shape, mediastinal contours and density of bone structures, which also might lower the performance of the current systems when applied to this population.

#### Tuberculosis and pneumonia

In the last few years, several studies have been published on the detection and diagnosis of consolidations in chest radiographs. Several groups have worked on AI to detect pulmonary tuberculosis in chest radiographs because resources and trained personal in developing countries are scarce, so automated evaluation of chest radiographs is especially beneficial in these countries. One of the first algorithms for tuberculosis, developed by Hogeweg et al. [33], showed a slightly lower performance than the observer for the detection of tuberculosis in a high-incidence population. Qin et al. [10] compared three available commercial deep learning systems and found similar performance of all systems for the detection of tuberculosis in chest radiographs, with an AUC ranging from 0.92 to 0.94 as compared with molecular testing. The AI systems reached higher specificity than the participating radiologists in the study, at a matched sensitivity level [10].

In addition to studies on AI for tuberculosis, studies are performed for AI that can help detect pneumonia [34–36]. In one of these studies [34], CheXNeXt, a convolutional neural network developed by the Stanford Machine Learning Group that can detect 14 pathologies on anteroposterior (AP) or posteroanterior (PA) chest radiographs, was tested on the chestX-ray8 dataset [37]. For 11 of the 14 pathologies, CheXNeXt achieved radiologist-level performance, including consolidations (AUC of 0.84 for radiologists versus 0.89 for the algorithm) [34]. In this study, radiologists achieved higher performance for the detection of emphysema, cardiomegaly and hiatus hernia [34].

Few studies have been performed in the pediatric population. The first study that was published examined AI to find any abnormality in pediatric chest radiographs in a very-high-incidence population suspected of tuberculosis (113/119 images) [38]. The system reached reasonable performance with an AUC of 0.78 for correctly identifying abnormal regions in the image [38].

A recent study reported results for CAD4Kids [39], which identified pneumonia in children younger than 5 years on chest radiographs. Sensitivity of the system was 76%, with a specificity of 80%. ROC performance was 0.85, which was significantly lower than that of a reference observer who reached an AUC of 0.98.

In the study by Tang et al. [40], the authors tested their algorithm for the detection of pneumonia on pediatric data and reached an AUC of 0.92, which was lower than the AUC of 0.98 on adult data. After retraining on pediatric data, the AUC improved to 0.98. And after fine-tuning, their algorithm improved further and reached an AUC of 0.99 for classifying normal images versus images with pneumonia [40].

Chen et al. [41] developed an algorithm for detecting common abnormalities in chest radiographs specifically for children ages 1–17 years. The reference standard in the study was set by a pediatric pulmonologist and radiologist in consensus. The algorithm reached an accuracy of 72% for detecting bronchopneumonia and 85% for detecting lobar pneumonia on a test set of 531 images [41]. The authors found greater disagreement in children younger than 5 years. This might be explained by a greater variance of chest shape in younger children.

Zucker et al. [42] explored the performance of an AI algorithm to determine the Brasfield score in children with cystic fibrosis. Their model, trained on roughly 1,800 children and tested on 200 children, achieved comparable performance with five participating radiologists for the most sub-features in scoring cystic fibrosis components in chest radiographs according to the Brasfield score [42].

Currently, seven certified AI products can detect pneumonia and five products can detect pulmonary tuberculosis. CAD4TB, a commercial AI system for the automated detection of tuberculosis, is the only certified product that can be used in children age 4 years and older [17].

#### Pneumothorax

Several studies have been performed for automatic pneumothorax detection in chest radiography, of which few have been developed into commercial software. Hwang et al. [6] reported excellent performance of their algorithm, with a median AUC of 0.97 (range 0.92–0.99), for the detection of pulmonary malignancies, tuberculosis, pneumonia and pneumothorax. Most radiologists improved in performance after using the algorithm.

Park et al. [43] tested a convolutional neural network to detect pneumothorax after transthoracic needle biopsy. Their network was trained on 1,596 chest radiographs and reached an AUC of 0.90 for the detecting pneumothorax 3 h after needle biopsy. As a reference standard, they used a consensus read of two experienced radiologists [43].

The study of Rajpurkar et al. [34] also evaluated pneumothorax detection. In this study their algorithm reached an AUC of 0.944, compared to an average AUC of 0.940 of radiologists. No statistical difference was found between the algorithm and the radiologists. Unfortunately, the algorithm was not tested on an external dataset.

Chen et al. [41], who developed a deep learning system for the most common abnormalities in pediatric chest radiographs, also tested their algorithm for the detection of pneumothorax and reached an accuracy 86%.

Currently, 11 commercially available packages offer pneumothorax detection. According to the reported literature, none of the certified products has been trained on pediatric data. Therefore, performance level of these AI products might be lower in the pediatric population.

#### Lines/tubes

Another possible worthwhile application is the detection of lines and tubes in chest radiographs. Malposition of lines disrupts proper treatment, and clear visualization of the line can help to avoid repeat radiographs and reduce patient radiation dose.

Several studies have been performed for the detection of lines and tubes in chest radiographs [44–49]. Generally the algorithms are quite good in classifying the presence versus absence of an endotracheal tube [50], but the systems perform worse when the exact position of the tip of the tube is sought.

Few studies for AI in the detection of lines and tubes have been performed in the pediatric population. In the study performed by Kao et al. [51], the authors developed an algorithm for detecting endotracheal tubes in neonatal chest radiographs. The authors evaluated the algorithm on 528 images with endotracheal tubes and 816 images without endotracheal tubes. The discriminant performance for detecting the existence of a tube reached an AUC of 0.94 [51]. The distance error of the tip of the tube was on average 1.89±2.01 mm [51].

In a study by Yi et al. [52], the authors used data with synthetic nasogastric tubes, endotracheal tubes and umbilical catheters to test their algorithm. The precision (i.e. true positives / [true positives + false positives]) for their algorithm was 0.80 [52]. According to the authors, their work can contribute to the development of a system that detects all lines and catheters in X-ray images and could be used to prioritize images that show malpositioned lines and request urgent review by the radiologist [52].

No commercial AI products are available that locate lines and tubes in chest radiographs. However, a single product on the market aims to optimize contrast for certain identification of lines and catheters (ClearRead Confirm; Riverain Technologies, Miamisburg, OH).

### Chest computed tomography

**Fig. 3:**
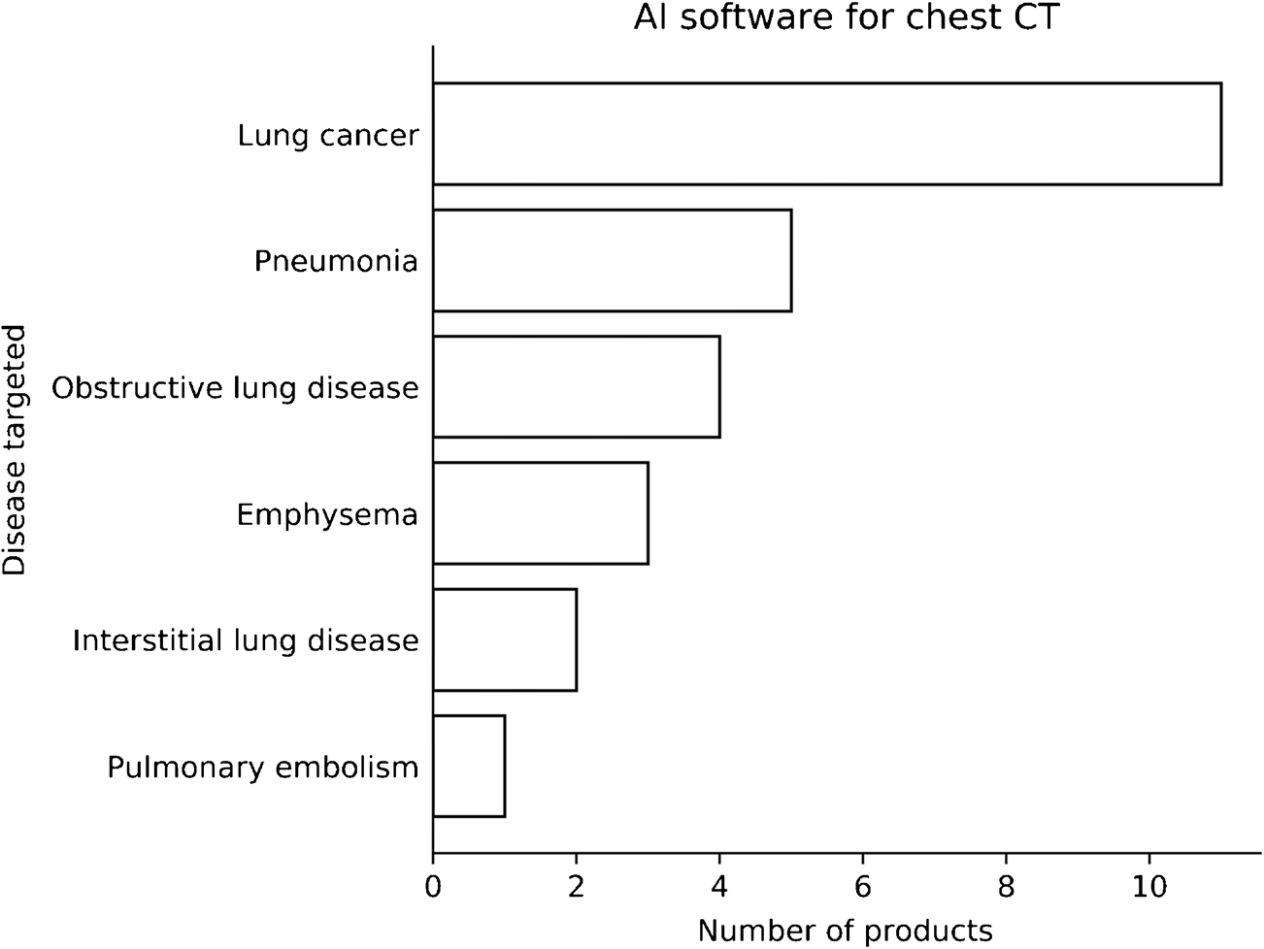
Bar chart shows artificial intelligence (AI) products available for chest CT at the time of this report [2]

Currently 21 CE-marked AI products for chest CT are available for use in daily clinical practice. Most applications focus on lung cancer detection and characterization. Other applications focus on the detection of pneumonia, especially in cases of coronavirus disease 2019 (COVID-19); the detection of obstructive lung disease; or the quantification of emphysema. Very few products are available for interstitial lung disease or the detection of pulmonary embolism (Fig. 3). Automatic detection of pulmonary embolism, quantification of emphysema, and detection and quantification of COVID-19 and fibrosis are less relevant for the pediatric population and therefore are not discussed here.

#### Lung nodules

In CT, automatic lung nodule detection is the most widely studied application. The introduction of lung cancer screening has accelerated the quality and number of products for lung nodule detection in CT. Several studies have shown outstanding performance for the detection of lung nodules in CT [26, 28, 53, 54]; however, characterization of the nodules is a bigger issue. Many of the found nodules are small and benign. This has induced several approaches to characterize nodules and automatically estimate each nodule’s risk of malignancy [55, 56]. A recent study showed significantly improved performance of a convolutional neural network model for risk assessment of small nodules in CT compared with the Brock model, which is currently used for malignancy risk assessment, with an AUC of 0.90 compared with 0.87, respectively [55].

Eleven AI products are available for detecting pulmonary nodules in chest CT with the goal of finding early lung cancer. In the pediatric population, assessing pulmonary nodules in chest CT is not a clinical issue, and none of the products has been developed for the pediatric population. Nonetheless, these AI products might be useful for detecting pulmonary metastases, which is applicable in a specific pediatric population; none of products that is available, however, is specifically designed to detect pulmonary metastases.

#### Obstructive lung disease

Four products on the market focus on obstructive lung disease. These include chronic obstructive lung disease, asthma and cystic fibrosis. The latter two, especially, are applicable to children. Although CT examinations in asthma are not commonly performed, CT in cystic fibrosis patients is used to monitor the disease. AI software can detect minor attenuation differences in the lung, and thereby detect areas of air-trapping. Further, bronchopathic changes such as bronchial wall thickening and bronchiectasis can be detected and quantified. DeBoer et al. [57] showed that the number of airway counts on inspiratory high-resolution CT and the percentage of low-attenuation density on expiratory CT were significantly higher in children with cystic fibrosis.

A few products that can assess airway changes and air-trapping are available and should be usable in people with cystic fibrosis. However, no studies have been performed to assess the value of these products over visual assessment. Recently, Thirona B.V. (Nijmegen, The Netherlands) [58] obtained a patent for quantitative CT analysis of cystic fibrosis in children. They aim to develop an algorithm to identify early changes on CT scans in children with cystic fibrosis.

#### Pneumothorax

Several studies have examined the use of artificial intelligence in chest CT for the detection of pneumothorax. One example is the quick identification of pneumothoraces on chest CT [59]. This application reached a sensitivity of 100% and a specificity of 82.5%. The authors suggested that the tool could be used for triaging and to notify the radiologist about the detected urgent findings [59]. Complicating factors might be the presence of emphysema or bullae that could erroneously be seen as a pneumothorax. Other researchers tried to automatically quantify the volume of the pneumothorax. Rohrich et al. [60] showed excellent performance of their algorithm, with a Pearson correlation coefficient of 0.996 between the automated quantification method and the manual measurement. For the pediatric population, one study investigated a computer-aided volumetry scheme for quantifying pneumothoraces in chest CT. The study performed by Cai et al. [61] also showed excellent performance, with Pearson correlation coefficient of 0.99 between the manual and automated measurements. This is important because a larger-volume pneumothorax might trigger different treatment. At this point, no commercial AI product is available for detecting and quantifying pneumothoraces in chest CT.

## Future directions

Although AI applications for thoracic radiology are one of the most developed in radiology, still the use of these applications in clinical practice is scarce. But, with recent improvements in quality of the products and more attention on the clinical issues rather than technical possibilities, more products are expected to be implemented in the clinic in the near future. Unfortunately, this does not mean that the current AI products are of added value for the pediatric population. Literature with performance measures of AI products in thoracic radiology for the use in the pediatric population is limited, and very few of the current applications could be used in the pediatric population. To develop AI in pediatric chest radiology, more studies are needed to evaluate the performance of current available systems and also to develop AI products specifically aimed at the pediatric population.

One approach is to extend existing algorithms aimed at adults to the pediatric population. However, the intended use of current AI products in radiology is tailored to adults. If new studies of existing AI products were to show reliable performance in the pediatric population, the intended use of these products would be different and new certifications needed.

Furthermore, developers should focus on clinical issues in pediatric chest radiology. For instance, detection of pneumonia or pulmonary metastases in children could be helpful in clinical practice. Pneumonia in the pediatric population is common and can easily be diagnosed with chest radiography. Accurate detection of pulmonary metastases in chest CT of children with known malignancy might improve patient outcome. Apart from extending existing products, other difficult radiographic use cases could be addressed, for instance the automated grading of infant respiratory stress syndrome in premature newborns. Also, some products could be geared toward the non-pediatric radiologist, who might be less experienced in assessing pediatric imaging because of less exposure, for instance during on-call hours. One example of this could be automated detection of lines and tubes in newborns, an application that would also be helpful for clinicians.

Finally, AI could be used to improve imaging at acquisition and lower the radiation dose. With AI, CT dose could be lowered while preserving image quality in children [62, 63]. AI could also be used to reduce artifacts [64, 65]. This topic is outside the scope of this review because our focus is on diagnostic applications; however, continuing improvements in applications can be expected. CT imaging in children might therefore be less harmful with a lower radiation dose.

## Conclusion

Multiple artificial intelligence products for chest radiology are available, covering a wide range of thoracic abnormalities. Reported performances of current products are promising, but little is known about their value in daily clinical practice. Products for pediatric chest radiology are scarce but might become more prevalent in the near future. The available studies and evidence discussed in this paper suggest that products for automated detection of pneumonia, tuberculosis and lines and tubes in chest radiography and cystic fibrosis in chest CT can be expected.
